# Development of a Novel Virus-Like Particle Vaccine Platform That Mimics the Immature Form of Alphavirus

**DOI:** 10.1128/CVI.00090-17

**Published:** 2017-07-05

**Authors:** Akane Urakami, Atsuko Sakurai, Momoko Ishikawa, Moh Lan Yap, Yevel Flores-Garcia, Yasunari Haseda, Taiki Aoshi, Fidel P. Zavala, Michael G. Rossmann, Sachiko Kuno, Ryuji Ueno, Wataru Akahata

**Affiliations:** a VLP Therapeutics, Gaithersburg, Maryland, USA; b Department of Biological Sciences, Purdue University, West Lafayette, Indiana, USA; c Department of Molecular Microbiology and Immunology, Johns Hopkins Bloomberg School of Public Health, Johns Hopkins University, Baltimore, Maryland, USA; d Vaccine Dynamics Project, BIKEN Innovative Vaccine Research Alliance Laboratories, Research Institute for Microbial Diseases, Osaka University, Suita, Osaka, Japan; Duke University Medical Center

**Keywords:** alphavirus, chikungunya virus, malaria, vaccines, virus-like particle

## Abstract

Virus-like particles (VLPs) are noninfectious multiprotein structures that are engineered to self-assemble from viral structural proteins. Here, we developed a novel VLP-based vaccine platform utilizing VLPs from the chikungunya virus. We identified two regions within the envelope protein, a structural component of chikungunya, where foreign antigens can be inserted without compromising VLP structure. Our VLP displays 480 copious copies of an inserted antigen on the VLP surface in a highly symmetric manner and is thus capable of inducing strong immune responses against any inserted antigen. Furthermore, by mimicking the structure of the immature form of the virus, we altered our VLP's *in vivo* dynamics and enhanced its immunogenicity. We used the circumsporozoite protein (CSP) of the Plasmodium falciparum malaria parasite as an antigen and demonstrated that our VLP-based vaccine elicits strong immune responses against CSP in animals. The sera from immunized monkeys protected mice from malaria infection. Likewise, mice vaccinated with P. yoelii CSP-containing VLPs were protected from an infectious sporozoite challenge. Hence, our uniquely engineered VLP platform can serve as a blueprint for the development of vaccines against other pathogens and diseases.

## INTRODUCTION

Virus-like particle (VLP) technology is a very powerful method for developing vaccines (1–3). VLPs mimic the conformation of authentic native viruses without being infectious, since they do not carry any viral genetic material. When presented within a host immune system, VLPs evoke effective immune responses without triggering the side effects associated with the native virus. Several VLP-based vaccines are on the market, including vaccines against hepatitis B virus and human papillomavirus (4). Furthermore, through genetic fusion or chemical conjugation, VLPs are attractive carrier proteins of foreign antigens, since they can efficiently display them within a host immune system (5–7). An important advantage of VLP-based vaccine platforms is that VLPs can present antigens in a dense, repetitive manner, thus effectively enabling the cross-linking of B cell receptors (BCRs) (8). Multivalent antigens bound to BCRs show more efficient processing and presentation than monovalent antigens (9). Cross-linking of BCRs by multivalent antigens leads a conformational change in BCRs and induces signaling-active BCR microcluster formation, leading to robust B cell activation and signaling (10). Therefore, VLP-based approaches offer great potential for developing effective vaccines against many pathogens, including antigens known to be weakly immunogenic.

The chikungunya virus (CHIKV) is a mosquito-borne alphavirus that causes chikungunya fever. The genome of the CHIKV is composed of a positive single-stranded RNA encoding four nonstructural (nsP1 to nsP4) and five structural (C, E3, E2, 6K, and E1) proteins. The five structural proteins are translated as a single polyprotein, from which the capsid (C) protein is cleaved off by capsid autoproteinase. The envelope polyprotein precursor (E3-E2-6K-E1) is translocated to the endoplasmic reticulum (ER) and further processed by host signalases, resulting in E1, 6K, and p62 (or the E3E2 precursor polyprotein). E1 and p62 are subsequently assembled as heterodimers in the ER and processed through the Golgi and *trans*-Golgi networks, where p62 undergoes a furin-dependent maturation cleavage. The resulting mature CHIKV virion contains 240 heterodimeric spikes of E2/E1 on its surface (11, 12). It is the expression of the structural proteins that gives rise to a chikungunya VLP (CHIK VLP), which has an icosahedral structure comprised of 240 copies of envelopes per particle arranged in a symmetric, repetitive array (triangulation number [T] = 4). CHIK VLPs, *per se*, have been shown to be a promising vaccine candidate against CHIKV: immunization with CHIK VLPs themselves induced a strong neutralizing antibody response against CHIKV in mice and monkeys and prevented viremia in monkeys after subsequent challenge with CHIKV (13). Furthermore, a phase I clinical trial demonstrated that CHIK VLP vaccine was safe and highly immunogenic (14).

In this study, by leveraging the solid safety profile and potent immunogenicity of CHIK VLPs, we created a novel vaccine platform derived from CHIK VLPs whereby foreign antigens were engineered for insertion into two positions within the surface-loop domains of the CHIKV envelope, resulting in 240 or 480 copies of antigen displayed on the CHIK VLP surface. This repetitive, highly symmetric array of antigens greatly enhances immune responses per the explanation on BCR multimeric cross-linking and downstream activation given above. Here, we describe the characteristics of our novel VLP vaccine platform and demonstrate its efficacy and applicability in the context of the malaria CSP antigen, a major surface antigen expressed on the surface of malaria sporozoites and involved in the initial infective stage by malaria parasites in humans (15). The central region of the P. falciparum malaria strain's CSP contains multiple tetrapeptide (Asn-Ala-Asn-Pro [NANP]) repeats. It has been reported that the NANP repeats are a B cell dominant epitope and that anti-NANP antibodies play an important role in protection from malarial infection (16). By incorporating the CSP repeat region as epitopes into our VLP platform, our vaccine induced high titers of anti-CSP antibodies in mice and monkeys and conferred protection against malarial infection in preclinical studies.

## RESULTS

### Development and characterization of CHIK VLP-based vaccine platform.

**FIG 1:**
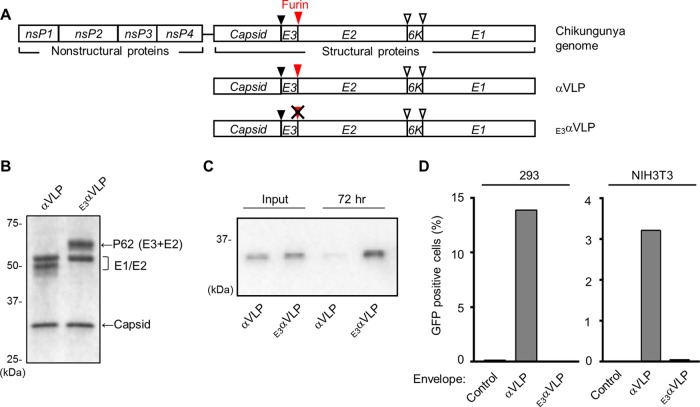
Development and characterization of αVLP vaccine platform. (A) Schematic representation of the CHIKV genome and of αVLP or _E3_αVLP expression vectors. Solid arrowhead, capsid autoproteinase cleavage site; open arrowheads, signal peptidase cleavage sites; furin; furin recognition sequence. (B) Purified αVLP and _E3_αVLP were separated by SDS-PAGE and visualized by Coomassie blue staining. A representative image from at least three replicates is shown. (C) αVLP or _E3_αVLP was added to the culture medium of adherent 293 cells and incubated for 72 h. The VLPs in the culture supernatant were detected by Western blotting with mouse anti-CHIKV antiserum. A representative blot of three independent experiments is shown. (D) Infectivity of the indicated pseudotyped lentiviral vectors in CHIKV-permissive cell lines. After incubation with pseudotyped lentiviral vectors for 72 h, GFP expression was assessed by flow cytometry. The results for one representative experiment of three performed are shown.

The expression of CHIKV structural proteins produces CHIK VLP in mammalian cells (13). First, we prepared an expression vector encoding CHIKV structural proteins (C-E3-E2-6K-E1) with a short linker introduced in E2, which will produce a VLP (here called αVLP) similar to the wild-type CHIK VLP (Fig. 1A, αVLP). During viral replication, E1 and p62 (or E3E2) are assembled as p62/E1 heterodimers and form 80 trimeric glycoprotein spikes. Eventually, p62 is cleaved into E3 and E2 by furin to produce the mature E2/E1 dimer, and E2 binds to receptors on the surface of a host cell, playing an important role in virus infection (11, 12, 17). It has been reported that the p62/E1 heterodimer is more acid resistant than the E2/E1 heterodimer and that the retention of E3 on the CHIK VLP enhances the stability of VLPs (18). We hypothesized that the E3 retained on CHIK VLP inhibits VLP binding and fusion to host cells and enhances the immunostimulatory activity of the vaccine platform. To test this hypothesis, we also created an expression vector for E3E2 cleavage-impaired αVLP (_E3_αVLP, E3 retained on αVLP) by mutating the furin recognition site (Fig. 1A, _E3_αVLP).

When transfected into 293F cells, the VLPs are secreted into the culture supernatant. We purified the VLPs and confirmed the protein expression using SDS-PAGE, followed by Coomassie blue staining. The results indicated the presence of capsid (30 kDa), E1 and E2 (both around 50 kDa) for αVLP, or E1 (50 kDa) and p62 (62 kDa) for _E3_αVLP (Fig. 1B). Next, we investigated the ability of these VLPs to enter cultured cells. Epithelium-derived cell lines, including human embryonic kidney 293 cells, are highly susceptible to CHIKV (19). αVLP- or _E3_αVLP-containing culture supernatant was added to the culture medium of adherent 293 cells, followed by incubation for 72 h. The culture medium was then clarified and analyzed for the levels of remaining VLP. Only _E3_αVLP was detected in the culture medium (Fig. 1C), suggesting that the retained E3 had prevented VLP entry into the cells. The entry of VLPs was further examined by using pseudotyped lentiviral vectors incorporating E2/E1 or p62/E1 glycoproteins. The pseudotyped lentiviral vector incorporating E2/E1 from αVLP showed infection of 293 cells and mouse fibroblast NIH 3T3 cells, cell lines that are permissive to CHIKV replication. In contrast, the pseudotyped vector incorporating p62/E1 from _E3_αVLP did not show infection of the cells (Fig. 1D), supporting our hypothesis that E3 prevents VLP entry into host cell.

### *In vivo* stability and distribution of αVLP and _E3_αVLP.

**FIG 2:**
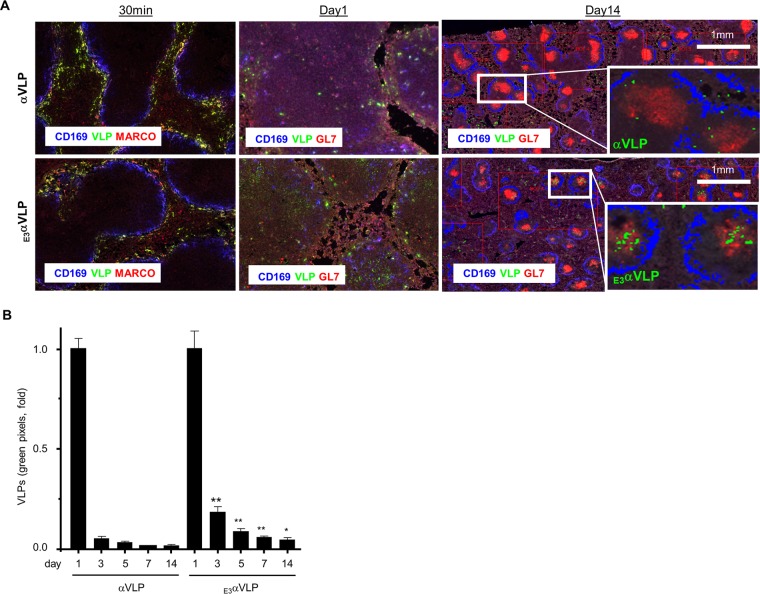
*In vivo* stability and distribution of αVLP and _E3_αVLP. (A) Splenic sections were stained for αVLP or _E3_αVLP (green), CD169 (blue), MARCO (red), and GL7 (red) over a 14-day time course. At each time point, the left panel shows αVLP, and the right panel shows _E3_αVLP. Representative images from three independent experiments are shown. (B) Quantitative analysis of VLP staining was performed on a pixel basis with five different spleen areas of the longitudinal section and normalized for αVLP or _E3_αVLP at day 1 staining as 1.0. Data are presented as means ± the standard errors of the means (SEM). Significance was evaluated for αVLP versus _E3_αVLP at the corresponding days by a Mann-Whitney test (*, *P* < 0.05; **, *P* < 0.01).

To examine these VLPs' stability and distribution *in vivo*, purified αVLP or _E3_αVLP was administered intravenously to mice, and the presence of VLPs in the spleen was assessed through staining with CHIKV-specific antibody (Fig. 2A). Both αVLP and _E3_αVLP (green) were trapped in marginal zone macrophages after 30 min (MARCO^+^, red). At day 1, αVLP was sporadically distributed, whereas _E3_αVLP preferentially accumulated in the B cell areas of the white pulp (inside CD169^+^, blue). At day 14, αVLP was almost undetectable; however, a significant amount of _E3_αVLP was retained in the germinal center (GC; GL7^+^, red). Quantitative image analysis over this time course further supported the long-lasting feature of _E3_αVLP *in vivo* (Fig. 2B). These results strongly suggest that the retained E3 enhanced VLP tissue retention in the GCs after administration compared to αVLP.

### Characterization of αVLPs inserted with malaria antigen.

**FIG 3:**
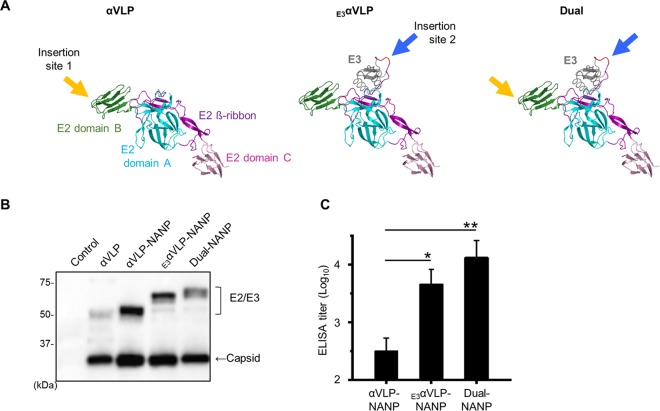
Characterization of αVLPs inserted with malaria antigen. (A) Structure of αVLP and _E3_αVLP envelope. Arrows indicate epitope insertion positions. (B) Western blotting of VLPs inserted with NP(NANP)_5_NA epitope. Expression vectors were transfected into 293F cells, and culture supernatants were tested for VLP expression on day 4. A representative image from at least three replicates is shown. (C) Immunogenicity in mice. BALB/c mice (*n* = 4) were immunized intramuscularly twice at 3-week intervals with 10 μg of the indicated VLPs with a NP(NANP)_5_NA epitope, and the serum anti-NANP antibody titer was determined by ELISA on day 31. Data are shown as mean endpoint titers ± the SEM. One-way ANOVA, followed by Dunnett's multiple-comparison test, was used to determine significance (*, *P* < 0.05; **, *P* < 0.01).

To test αVLP's capacity to accommodate foreign antigens, we identified two potential antigen insertion sites located in the surface loop region of CHIKV envelope based on the crystal structure of CHIKV (12). A foreign antigen can be inserted within E2 of αVLP (Fig. 3A, insertion site 1) or into the mutated furin cleavage site of _E3_αVLP (Fig. 3A, insertion site 2). When transfected into 293F cells, the inserted antigen and the viral envelope are expressed as a fusion protein and self-assemble into VLPs. As described in the introduction, CHIK VLP is comprised of 240 copies of E2/E1 heterodimeric envelopes per particle. Therefore, 240 copies of foreign antigen will be displayed on the αVLP surface in a symmetrical manner, which is known to enhance B cell signaling and induce high antibody titers (2, 20). By utilizing this platform, we developed new malaria vaccine candidates, which display the central NANP repeat region of CSP of the P. falciparum CSP as the epitope on the VLPs. CSP is the major surface antigen displayed during the infective stage of malaria and has been recognized as a likely target for malaria vaccine development (16, 21). The repeat region is highly conserved among different strains of P. falciparum, and it is considered an immunodominant B cell epitope (22). We inserted five NANP repeats into the cloning sites of αVLP (αVLP-NANP) and _E3_αVLP(_E3_αVLP-NANP). We also constructed Dual-NANP, which has the NANP repeat epitope in both insertion sites (Fig. 3A, Dual), thus displaying 480 copies of five NANP repeats per VLP. The NANP-inserted VLP expression was determined by Western blotting directly in the culture supernatants using anti-CHIKV antiserum (Fig. 3B). Mice were injected with the VLPs, and serum anti-NANP antibody titer was assessed by enzyme-linked immunosorbent assay (ELISA). Dual-NANP elicited the highest antibody titer among the three VLPs, and _E3_αVLP-NANP induced a significantly higher titer compared to αVLP-NANP (Fig. 3C). The endpoint titers (determined as described in Materials and Methods) were as follows: αVLP-NANP, 418; _E3_αVLP-NANP, 8,044; and Dual-NANP, 28,264.

### Structure and immunogenicity of VLPM01, an αVLP-based malaria vaccine candidate.

**FIG 4:**
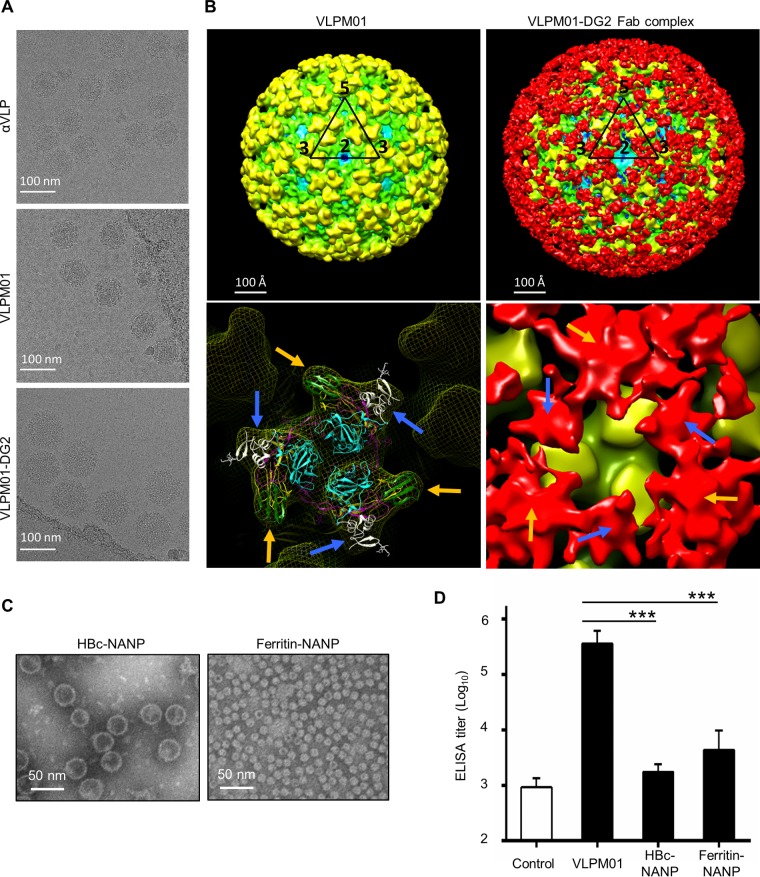
Characterization of the αVLP-based malaria vaccine candidate VLPM01. (A) Representative EM images of control αVLP, VLPM01, and VLPM01 complexed with the DG2 Fab fragments. (B) Cryo-EM reconstructions of VLPM01. (Upper left) 3D cryo-EM map of VLPM01, viewed down an icosahedral twofold axis. The black triangle marks the boundary of an icosahedral asymmetric unit. The numbers in black show the locations of the icosahedral two-, three-, and fivefold axes around the asymmetric unit. (Bottom left) Closer view of the icosahedral threefold trimeric spike in VLPM01 fitted with the crystal structure of CHIK p62-E1 (3N40). Gold arrows point to the insertion site at E2, and blue arrows point to the insertion site at E3. (Upper right) 3D cryo-EM map of VLPM01 complexed with the DG2 Fab fragments. (Bottom right) Trimeric spike on the icosahedral threefold axis of VLPM01 complexed with Fab fragments of DG2. Gold arrows point to the DG2 Fab density bound on the insertion site at E2. Blue arrows point to the DG2 Fab density bound on the insertion site at E3. (C) Representative transmission electron microscopic images of HBc-NANP and ferritin-NANP. (D) BALB/c mice (*n* = 4) were intramuscularly injected with 10 μg of control αVLP (no insert), VLPM01 [with NP(NANP)_13_NA inserted], HBc-NANP [with NANPNVDP(NANP)_3_ inserted], or ferritin-NANP [with NP(NANP)_13_NA inserted], and anti-NANP antibody titer was determined by ELISA on day 14. Data are shown as mean endpoint titers ± the SEM. One-way ANOVA, followed by Dunnett's multiple-comparison test, was performed to determine significance (***, *P* < 0.001).

Next, we optimized the length of the NANP repeat epitope. We evaluated the immunogenicity of the αVLPs inserted with different numbers of NANP repeats and found that the Dual-NANP inserted with 13 NANP repeats (here called VLPM01) induced high titer anti-NANP antibodies without sacrificing VLP yield (see Fig. S3 in the supplemental material). Examination of the purified αVLP and VLPM01 by electron microscopy (EM) demonstrated that the VLPs have similar morphology as the native virus (Fig. 4A, top and middle). Three-dimensional cryo-electron microscopy (3D cryo-EM) reconstruction of VLPM01 showed that it has a diameter of 67 nm and a T=4 quasi-icosahedral symmetry, which is similar to the previously described CHIK VLP structure and other alphaviruses (Fig. 4B, left). A 3D cryo-EM reconstructed map of VLPM01 complexed with the fragment antigen-binding (Fab) region of anti-NANP repeat monoclonal antibody (clone DG2) showed that NANP epitopes in both insertion sites were exposed on the surface of the VLPM01 particles and were accessible to the binding of Fab fragments (Fig. 4B, right). The EM image of VLPM01-DG2 complex is shown in Fig. 4A (bottom).

We also tested the immunogenicity of VLPM01 in mice and compared the antibody response with other nanoparticles displaying NANP epitopes. We have chosen two self-assembling protein nanoparticle platforms, hepatitis B virus core protein (HBc) and ferritin. HBc self-assembles into VLP which possesses strong immunogenic properties, and HBc VLP has been used extensively to present epitopes in the design of potential vaccines (23). Among those, HBc VLP displaying the central repeat region of P. falciparum CSP [NANPNVDP(NANP)_3_] has been reported as malaria vaccine candidate ICC-1132. We created HBc VLP with the CSP repeat epitope inserted and produced it in Escherichia coli as previously described (24). Ferritin is an immunogenic VLP-like nanoparticle, and it has been demonstrated that genetically engineered ferritin nanoparticles can present a multivalent array of various virus antigens (25, 26). We inserted the same number of NANP repeat as in VLPM01 into ferritin and produced it in 293F cells. EM confirmed that HBc-NANP and ferritin-NANP formed particles 31 to 40 nm and 12 to 16 nm in diameter, respectively (Fig. 4C). Mice were immunized with control VLP (αVLP without NANP epitope), VLPM01, HBc-NANP, or ferritin-NANP, and the anti-NANP antibody titer was measured. The average endpoint titer (final dilution) of VLPM01-immunized mice was 563,489, which was significantly higher than the titers for the other groups (Fig. 4D). The endpoint titers were as follows: HBc-NANP, 2,017; ferritin-NANP, 12,809; and control VLP, 1,157. These results suggest that the αVLP vaccine platform is very efficient at inducing immune responses against the displayed epitope.

### αVLP-based vaccine protected mice from malaria challenge.

**FIG 5:**
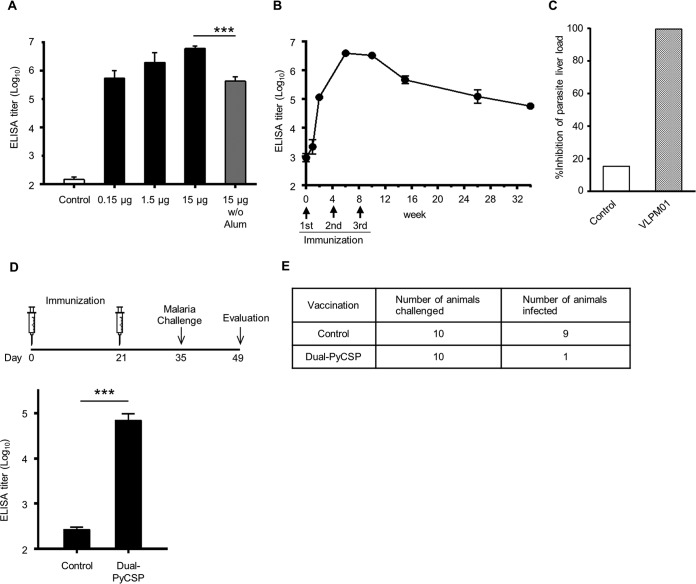
αVLP-based vaccine protected mice from malaria challenge. (A) BALB/c mice (*n* = 5) were immunized intramuscularly with the indicated doses of VLPM01 with or without alum three times at 3-week intervals. At 2 weeks after the last immunization, the anti-NANP antibody titer was determined by ELISA. Data are shown as mean endpoint titers ± the SEM. A Student *t* test was performed (***, *P* < 0.001). (B) Rhesus macaques (*n* = 4) were immunized intramuscularly with 45 μg of VLPM01 with alum three times at 4-week intervals. Serum anti-NANP antibody titer at various time points was determined by ELISA. Data are shown as mean endpoint titers ± the SEM. (C) B6 mice (*n* = 5) were injected intravenously with 450 μl of control or VLPM01-immunized monkey serum and then challenged intravenously with P. berghei/P. falciparum full CSP sporozoites. Liver parasite burdens were assessed at 40 h postchallenge by qPCR for P. berghei-specific 18S rRNA. The results are shown as the percent inhibition compared to naive, infected mice. (D) P. yoelii challenge experiment. BALB/c mice (*n* = 10) were immunized intramuscularly with 20 μg of control αVLP (no insert) or Dual-PyCSP [with (QGPGAP)_14_ inserted] with alum twice at 3-week intervals. Anti-PyCSP repeat antibody titer was measured 12 days after the second immunization. Data are shown as mean endpoint titers ± the SEM. A Student *t* test was performed (***, *P* < 0.001). (E) Malaria infection was determined by a Giemsa-stained thin blood smear procedure 14 days after the challenge.

The immunogenicity of VLPM01 was further tested with three different doses, together with alum adjuvant (aluminum hydroxide), in mice. The lowest dose of 0.15 μg was clearly immunogenic, and alum enhanced the anti-NANP antibody response compared to the use of VLPM01 alone (Fig. 5A). Next, we immunized rhesus macaques with 45 μg of VLPM01 with alum. A single immunization of VLPM01 stimulated the production of anti-NANP antibodies, and the immune response was enhanced by booster immunizations. The serum anti-NANP titer was maintained at high levels 6 months after the third immunization (Fig. 5B). The estimated anti-NANP antibody concentrations in the immunized monkey sera were 258 μg/ml (geometric mean anti-NANP antibody titer of 79,503 defined as the dilution factor to give an optical density [OD] of 1.0) and 231 μg/ml (70,655 defined as the dilution factor to give an OD of 1.0) after the second and third immunizations (measured at weeks 6 and 10), respectively.

To further examine the applicability of the αVLP vaccine platform, we assessed the protective efficacy of αVLP-based CSP vaccines using two malaria challenge models. First, we intravenously injected mice with the serum from an unimmunized or a VLPM01-immunized monkey and challenged the animals with murine malaria P. berghei sporozoites expressing a functional P. falciparum CSP (P. berghei/P. falciparum full sporozoites) (27). Liver parasite burdens were assessed 40 h postchallenge by quantitative PCR (qPCR) for P. berghei-specific 18S rRNA. The mice administered VLPM01-immunized monkey serum showed a >99.5% reduction in liver parasite burden (Fig. 5C and see Fig. S4 in the supplemental material), suggesting that the humoral responses induced by VLPM01 are sufficiently potent to prevent the liver-stage development of P. falciparum. Next, we prepared Dual-PyCSP, an αVLP with 14 QGPGAP repeat sequences from CSP of rodent malaria parasite P. yoelii inserted. Mice were immunized with two doses of control αVLP or Dual-PyCSP, and anti-PyCSP repeat antibody production was confirmed by ELISA (Fig. 5D). The mice were challenged intravenously with infectious P. yoelii sporozoites, and parasitemia was determined by a Giemsa-stained thin blood smear procedure on day 14 postchallenge. In the control αVLP-immunized group, 9 of 10 mice were infected with malaria parasites. In contrast, only one mouse was infected in the Dual-PyCSP-immunized group, yielding 89% vaccine efficacy (Fig. 5E). These results were also confirmed by reverse transcription-PCR analysis of P. yoelii-specific 18S rRNA at 14 days postchallenge in livers (data not shown). Taken together, the humoral immune responses induced by our αVLP-based vaccines confer significant protection against malaria infection.

## DISCUSSION

VLPs can function as molecular scaffolds for presenting foreign antigens and, due to their structural similarity to native viruses, VLPs can efficiently stimulate host immune cells. VLPs can serve as highly proficient tools for displaying antigens because their repetitive surface patterns and particulate structure induce potent immune responses. In addition, VLP-based vaccines have good safety histories and can be produced at large scales in heterologous expression systems (4). In this study, we created and characterized a novel vaccine platform based on CHIK VLP. Our data demonstrate that our αVLP vaccine platform can elicit high titer antibody responses against the inserted foreign antigen in mice and monkeys.

We developed wild-type αVLP and E3E2 cleavage-impaired _E3_αVLP and compared their characteristics *in vitro* and *in vivo*. Notably, _E3_αVLP which retains E3 on the particle was detectable longer *in vivo* and preferentially associated with GCs in the lymphoid organs. It is possible that the retained E3 stabilizes the VLP, enabling _E3_αVLP to remain in contact with B cells in GCs longer than αVLP (Fig. 2B). GCs are sites where high-affinity memory B cells are generated (28), and the sustained presence of _E3_αVLP can effectively promote immune responses. This is supported by evidence of _E3_αVLP-NANP inducing a significantly higher antibody titer against the inserted antigen compared to the furin-sensitive, wild-type αVLP-NANP (Fig. 3C). The density of antigens is important for B cell activation. Like native CHIKV, αVLP's quasi-crystalline surface with repetitive antigen array can be easily recognized by B cells. As shown in Fig. 4B, 480 copies of inserted antigens are displayed on the particle surface and bind to Fab. The distances between Fabs on the neighboring spikes are 10 to 15 nm, which is within the wingspan of typical IgG molecules (10 to 20 nm), suggesting that αVLP presents epitopes at optimal distances to facilitate BCR activation by bivalent engagement.

To assess the utility of our αVLP vaccine platform, we focused on the NANP repeat antigenic region of the CSP surface protein of P. falciparum. CSP of P. falciparum has been extensively studied as a promising malaria vaccine target. It has been reported that soluble recombinant P. falciparum CSP was highly immunogenic, and the immunogenicity was further enhanced by presenting CSP antigen on nanoparticles and VLPs (27, 29). Our αVLP-based malaria vaccine, VLPM01, successfully elicited high levels of anti-CSP NANP repeat antibody. The humoral immune responses induced by VLPM01 conferred protection against malaria infection. The immune response was maintained 6 months after the last booster immunization in monkeys (Fig. 5B). In addition to the anti-NANP antibodies, VLPM01 also induces antibodies against CHIKV backbone. There is a possibility that immunization with our αVLP may induce suboptimal levels of anti-CHIKV antibodies, leading to an increase in susceptibility to future CHIKV or cross-reactive alphavirus infections due to antibody-dependent enhancement (ADE) phenomenon (30, 31). There are no precise studies to predict the potential for ADE stemming from our alphavirus VLP vector; therefore, preclinical and clinical studies regarding ADE effects are needed.

Malaria is a serious public health problem worldwide, with 3.2 billion people at risk for malarial infection (32). Since the malaria parasite has a complex life cycle with multiple stages that unfold in human and mosquito hosts, vaccine development has been challenging. The most advanced malaria vaccine to date is RTS,S/AS01, developed by GlaxoSmithKline, which received a favorable opinion from the European Medicines Agency in 2015 (33). RTS,S/AS01 blocks the first stages of malaria infection by targeting CSP, and anti-NANP antibodies induced by the vaccine are likely to be one of the major mechanisms of protection in humans. It has been shown that the administration of anti-NANP monoclonal antibodies derived from an RTS,S/AS01 vaccine recipient conveyed protection against P. falciparum challenge in mice (34).

In our study, rhesus macaques were immunized with VLPM01 plus alum adjuvant. VLPM01 induced geometric mean anti-CSP antibody titers of 79,502 and 70,655 (defined as the dilution factor necessary to give an OD of 1.0) after the second and third immunizations, respectively. In contrast, the RTS,S plus AS adjuvants induced less than 20% as much total antibody titer in rhesus macaques (35). Although we cannot directly compare these results, the stark difference in immunogenic response levels suggests that our VLPM01 can induce higher concentrations of anti-CSP antibodies than RTS,S. An association between anti-CSP antibody titers and protection was observed in several clinical trials, with the average titers of protected volunteers being 25 to 250 μg/ml (15). The estimated anti-NANP antibody concentration in our VLPM01-immunized monkey serum was >200 μg/ml; therefore, we believe that VLPM01 is a promising malaria vaccine candidate.

A caveat to the use of VLPs as a vaccine platform is the potential for reduced immunogenicity stemming from any preexisting immunity against the derivative virus or other cross-reactive viruses to the said VLP. Although this was the case for some viral vaccine vectors, it has also been reported that antibody responses against herpes simplex virus- or poliovirus-vectored antigens were not affected by prior immunities (36). The isolation and characterization of human monoclonal antibodies from CHIKV-infected and recovered individuals (37) and the screening of a panel of mouse and human monoclonal antibodies against CHIKV (38) revealed that the dominant neutralizing epitopes are located in E2 protein of CHIKV. In VLPM01, those epitopes are partially masked by the retained E3 or disrupted by the inserted NANP epitopes. Therefore, we expect that preexisting anti-CHIKV neutralizing antibodies induced by natural CHIKV infection may have minimal impact on VLPM01's vaccine immunogenicity.

Although more studies are needed on the effects of preexisting immunity, our preclinical data convincingly demonstrate that our αVLP vaccine bears unique and promising advantages as a new generation vaccine platform. First, by engineering two insertion sites, αVLP can display two different antigens of choice at 240 dense copies each. Second, our αVLP can display relatively large antigens depending on their structure. Combined with the good safety profile and generally robust immunogenicity induced by VLPs, our αVLP technology holds the promise of serving as a powerful template for the development of next-generation vaccines against many other pathogens and diseases.

## MATERIALS AND METHODS

### Vector construction.

The VLP expression constructs and the αVLP-based vaccine candidates used in this study are illustrated in Fig. S1 in the supplemental material. A gene coding for CHIKV strain 37997 structural proteins (C-E3-E2-6K-E1) was synthesized by Blue Heron (Bothell, WA) and cloned into pUC119-derived vector under the control of human cytomegalovirus early-immediate promoter. The plasmid encoding αVLP was created by inserting BspEI and BamHI restriction sites as a Ser-Gly-Gly-Gly-Gly-Ser linker between amino acids (aa) 206 and 207 in the E2 domain. The plasmid encoding _E3_αVLP was prepared by replacing the furin cleavage site (E3 aa 61 to 64) with the Ser-Gly-Gly-Gly-Gly-Ser linker. SacII restriction site was introduced without changing the amino acid sequence to the E2 domain (aa 197 to 198) to create dual-epitope constructs. αVLP-NANP and _E3_αVLP-NANP were prepared by inserting the GNP(NANP)_5_NAG sequence into the BspEI/BamHI cloning sites of αVLP or _E3_αVLP, respectively. Dual-NANP was prepared by replacing NotI/SacII region of αVLP-NANP with NotI/SacII fragment of _E3_αVLP-NANP. The epitopes for other constructs used were as follows: VLPM01, GNP(NANP)_13_NAG; and Dual-PyCSP, G(QGPGAP)_14_G. The gene encoding ferritin-NANP was created as follows and cloned into pUC59. The leader sequence of human IL-2 (aa 1 to 19), followed by the Ser-Gly-Gly-Gly-Gly-Ser linker and Helicobacter pylori non-heme-iron-containing ferritin (aa 5 to 167; GenBank WP_000949190) with a point mutation (N19Q) (25), was synthesized by GeneArt (Thermo Fisher Scientific, Waltham, MA). The NANP epitope, GNP(NANP)_13_NAG, was inserted into the BspEI/BamHI cloning sites within the linker. HBc-NANP was created based on the previously reported malaria vaccine candidate (24). The NANP epitope, NANPNVDP(NANP)_3_ modified by the addition of 5′ BspEI and 3′ BamHI cloning sites, was inserted between aa 78 and 79 of the truncated HBc (aa 1 to 149). The gene encoding HBc-NANP was synthesized by GeneArt and cloned into pET-30a vector (EMD Millipore, Billerica, MA).

### Cell lines.

FreeStyle 293F cells were purchased from Thermo Fisher Scientific and cultured in suspension in serum-free FreeStyle 293 expression medium at 37°C in the presence of 8% CO_2_. The cells were authenticated by short tandem repeat profiling and confirmed to be free of mycoplasma contamination by using a LookOut mycoplasma PCR detection kit (Sigma-Aldrich, St. Louis, MO). 293T/17, 293, and NIH 3T3 cells were obtained from the American Type Culture Collection (ATCC; Manassas, VA). 293 cells (293F cells in CD293) were obtained from Thermo Fisher Scientific. The cells were cultured in Dulbecco modified Eagle medium (Sigma-Aldrich) containing 10% fetal bovine serum and penicillin-streptomycin solution (Thermo Fisher Scientific) at 37°C in the presence of 5% CO_2_.

### Antibodies against CHIKV and CSP-NANP.

Mouse and rabbit anti-CHIKV antisera were obtained from animals immunized with 20 μg of CHIK VLP three times at 3-week intervals and characterized by immunoblotting and immunohistochemistry (see Fig. S2A and B in the supplemental material). Anti-NANP mouse monoclonal antibody (clone DG2) was generated from a female BALB/c mouse immunized with Dual-NANP with Ribi adjuvant (Sigma-Aldrich). At 4 days after the last inoculation, the spleen was aseptically removed for hybridoma preparation according to standard procedures (39). Hybridomas producing antibodies that recognize NANP peptides but do not react with CHIK VLP were screened by ELISA and further cloned. The supernatants of hybridoma cultures were collected, clarified by centrifugation, and filtered through a 0.45-μm-pore-size membrane filter. The antibody was purified by using an rProtein A/Protein G GraviTrap kit (GE Healthcare Life Sciences, Pittsburgh, PA) according to the manufacturer's instructions and characterized by immunoblotting (see Fig. S2C in the supplemental material).

### Production and purification of VLPs.

The VLPs except for HBc-NANP were produced in FreeStyle 293F cells. The cells were transfected with VLP-expressing plasmids by using GeneX Plus transfection reagent (ATCC) according to the manufacturer's instructions. At 4 days after transfection, the cell culture supernatant was harvested and clarified by centrifugation and filtration with 0.45-μm-pore size polyethersulfone (PES) membrane. The VLPs secreted in the culture supernatant were collected by using OptiPrep density gradient medium (Sigma-Aldrich) as described previously (13) and further purified by using a HiPrep 16/60 Sephacryl S-500 HR column (GE Healthcare Life Sciences). The eluates containing purified VLPs were concentrated by Amicon Ultra-15 centrifugal filter units (EMD Millipore) and filtered with a 0.20-μm-pore-size PES membrane. HBc-NANP VLPs were expressed in E. coli and purified by ammonium sulfate precipitation and column chromatography. The E. coli strain, DH5α, was transformed with pET-HBc-NANP plasmid, and a single colony was cultured. VLP expression was induced by adding 0.1 mM IPTG (isopropyl-β-d-thiogalactopyranoside), and the cells were harvested, resuspended in TSE buffer (20 mM Tris-HCl [pH 7.9], 150 mM NaCl, 1 mM EDTA), and sonicated. Soluble lysate was collected by centrifugation and, after ammonium sulfate precipitation, the VLPs were purified using a combination of Superdex 200 and MonoQ columns (GE Healthcare Life Sciences). The eluates containing purified VLPs were pooled, concentrated, and dialyzed in a buffer containing 50 mM Tris-HCl (pH 7.4), 50 mM NaCl, and 1 mM EDTA. The purified VLPs were separated by SDS-PAGE and stained with InstantBlue Coomassie protein stain (Expedeon, San Diego, CA).

### VLP entry into 293 cells.

293 cells were maintained as adherent monolayer cultures, plated on a 12-well culture plate at 10^5^ cells/well, and cultured overnight. The VLP-containing culture supernatants were added to the well and, after 72 h, the conditioned medium was collected and analyzed for the remaining VLPs by Western blotting with mouse anti-CHIKV antiserum.

### Western blotting.

VLP-containing culture supernatant was separated by SDS-PAGE, and the electrophoresed proteins were transferred onto nitrocellulose membranes and blotted with mouse anti-CHIKV antiserum. After incubation with horseradish peroxidase (HRP)-conjugated goat anti-mouse IgG (Santa Cruz Biotechnology, Dallas, TX), membranes were developed using Clarity ECL Western blot substrate (Bio-Rad Laboratories, Hercules, CA).

### Production of pseudotyped lentiviral vectors and cell entry analysis.

We created plasmids expressing glycoproteins E1/E2 and p62/E1 from αVLP and _E3_αVLP expression vectors. Other lentiviral plasmids were purchased from Cell Biolabs (San Diego, CA). 293T/17 cells were seeded onto a 10-cm dish at a density of 2 × 10^6^ cells one day before transfection. The cells were transfected with 4 μg of glycoprotein expression plasmid, 12 μg of a transducing vector encoding a GFP reporter gene (pLenti-GFP), 4 μg of a packaging plasmid that expresses all HIV-1 structural proteins except Env and Rev (pCgpV), and 4 μg of a Rev expression vector (pRSV-REV) using Lipofectamine 2000 reagent (Thermo Fisher Scientific). Empty vector (pUC19) served as negative control. Packaged lentivirus-containing supernatant was collected by centrifugation and filtered through a 0.22 μm membrane. 293 cells and NIH 3T3 cells were seeded onto 24-well plates at 10^5^ cells/well one day before infection. The cells were infected with pseudotyped lentiviral vector with Polybrene at 8 μg/ml. The culture medium was changed to fresh medium after 1 day. At 3 days postinfection, green fluorescent protein (GFP) expression was analyzed by flow cytometry using an Attune flow cytometer (Thermo Fisher Scientific).

### Distribution of VLPs *in vivo*.

The experiments were conducted with the approval of the Animal Care and Use Committee of the Research Institute for Microbial Diseases, Osaka University. Then, 40 μg of αVLP or _E3_αVLP was intravenously administered into the tail veins of 6-week-old female BALB/c mice in a volume of 200 μl of phosphate-buffered saline (PBS). After the indicated time points, mice were sacrificed, and the spleens were removed and processed for cryosections as described previously (40, 41). Cryosections (6 μm) were fixed with 4% paraformaldehyde in PBS (pH 7.2) at 4°C for 5 min, blocked with StartingBlock blocking buffer (Thermo Fisher Scientific) for 10 min, and then sequentially incubated with primary antibodies and fluorescent-dye-conjugated secondary antibody and streptavidin. Three independent experiments (*n* = 5, 10, and 12) were performed. The antibodies and reagents used for staining were as follows: rat anti-mouse/human GL7 (phycoerythrin conjugated, GL7; BioLegend, San Diego, CA), rat anti-mouse MARCO (phycoerythrin conjugated, ED31; Bio-Rad Laboratories), rat anti-mouse CD169 (biotin conjugated, MOMA-1; BMA Biomedicals, Augst, Switzerland), rabbit anti-CHIKV antiserum and goat anti-rabbit IgG (fluorescein isothiocyanate conjugated; Sigma-Aldrich), and streptavidin (brilliant violet 421; BioLegend). Whole-section four-color fluorescent images were obtained with tiling options by Olympus IX83 inverted microscope system with a DP80 digital camera and a 10× objective lens (UPlanFL N, NA = 0.30; Olympus, Tokyo, Japan). All data were acquired and analyzed by using cellSens Dimension software (Olympus).

### Immunizations and measurement of anti-CSP antibody titer.

Immunization and serum sample preparation were conducted at Bioqual, Inc. (Rockville, MD). Female BALB/c mice were purchased from Harlan (Frederick, MD), and male and female Indian-origin rhesus macaques were purchased from PrimGen (Hines, IL). Mice were 7 to 11 weeks old and rhesus macaques were 5 to 8 years old at the time of the experiments. All animal experiments were conducted once under Institutional Animal Care and Use Committee-approved and Office of Laboratory Animal Welfare-assured conditions. Serum was collected from individual animals, and the anti-CSP repeat antibody titer was measured by ELISA. The ELISA plates were coated overnight with (NANP)_6_ peptides for P. falciparum or (QGPGAP)_4_ peptides for P. yoelii at 100 ng/well. Plates were blocked with 5% skim milk solution for 1 h. Samples were serially diluted in 5% skim milk and incubated in coated wells for 1 h, followed by 1 h of incubation with HRP-conjugated goat anti-mouse or anti-monkey antibody (Santa Cruz Biotechnology). Wells were developed using SureBlue TMB microwell peroxidase substrate (KPL, Gaithersburg, MD) and read on a microplate reader (BioTek, Winooski, VT). The endpoint titers of serum antibodies against (NANP)_6_ peptides or (QGPGAP)_4_ peptides were determined as the serum dilution that gives an optical density of 3 standard deviations above the negative control. The serum samples collected from VLPM01-immunized monkeys were also evaluated at the Walter Reed Army Institute of Research (WRAIR) International Reference Center for Malaria Serology (Silver Spring, MD). Anti-NANP antibody ELISA titers were measured by using anti-human IgG-HRP as a secondary antibody. The antibody titers were initially defined as the serum dilution yielding an optical density of 1.0 in a standardized assay and then converted to μg/ml concentrations. The investigators were not blinded to allocation of samples during these experiments and outcome assessment.

### Electron microscopy, cryo-EM, and image analysis.

The morphologies of HBc-NANP VLPs and ferritin-NANP nanoparticles were analyzed at the Johns Hopkins School of Medicine Microscope Facility (Baltimore, MD). Briefly, the VLPs were fixed in 4% formaldehyde, and fixed samples were placed on glow-discharged carbon-coated 200-mesh copper grids. The grids were then stained with 1% phosphotungstic acid and visualized by Philips CM120 transmission electron microscopy at 80 kV with an AMT XR80 8 megapixel camera. For the cryo-EM analysis of VLPM01, aliquots of 2.5 μl of sample at a 3-μg/ml concentration were loaded on glow-discharged C-Flat grids (CF-2/2-4C-50). These grids were blotted for 5 s and flash frozen in liquid ethane using a Gatan CP3 plunge freezer. The grids were viewed using the Purdue University's FEI Titan Krios electron microscope operated at 300 kV. Images were recorded with a Gatan K2 Summit detector calibrated to have a magnification of 38,461, yielding a pixel size of 0.65 Å. A total dose of 36 e^−^/Å^2^ and an exposure time of 7.6 s were used to collect 38 movie frames. Fully automated data collection was implemented using LEGINON (42). VLPM01 particles were incubated with Fab fragments of neutralizing mouse monoclonal antibody DG2 against the NANP repeats at 4°C for 30 min using a stoichiometric ratio of about two Fab fragments per insertion site. Grids were prepared as described for VLPM01, and images were recorded under the same conditions. A total of 2,800 particles each of VLPM01 and VLPM01 complexed with DG2 were boxed using the EMAN2 package (43). MOTIONCORR software (44) was used to correct the beam-induced motion. Contrast transfer function parameters were estimated using CTFFIND3 (45). The 2D classifications were performed using RELION (46), and the 3D reconstructions were performed using jspr software (47). The final reconstructions were estimated to be 11 and 12 Å, respectively, based on the gold-standard Fourier shell correlation criterion of 0.143 (48). Both maps were low-pass filtered to a 20-Å resolution. Both maps have been deposited in the Electron Microscopy Data Bank (www.emdatabank.org) under EMDB accession codes EMD-8424 and EMD-8425, respectively.

### Passive transfer of immunoglobulin and challenge in mice.

Five- to eight-week-old female C57BL/6 mice were purchased from the National Cancer Institute (Fredrick, MD) and housed in the animal care facility at Johns Hopkins University. All procedures were performed in accordance with the National Institutes of Health standards, as set in the Guide for the Care and Use of Laboratory Animals. Mice were sorted into different groups and injected intravenously with control or VLPM01-immunized monkey serum (450 μl/mouse) and immediately challenged intravenously with 2,000 P. berghei transgenic sporozoites expressing the full P. falciparum CSP. The serum samples were coded, and the experiment was conducted by investigators blinded to the group allocation. The VLPM01-immunized monkey serum was collected weekly between weeks 5 and 8 from a monkey injected with 45 μg of VLPM01 with alum twice and pooled. After 40 h, the livers were harvested, and RNA was isolated to quantify the P. berghei-specific 18S rRNA level by qPCR as previously described (49).

### Mouse malaria challenge model.

Six- to eight-week-old female BALB/c mice were immunized intramuscularly with 20 μg of control αVLP or Dual-PyCSP with alum adjuvant (Alhydrogel; Sergeant Adjuvants, Clifton, NJ) on days 0 and 21. Serum anti-PyCSP repeat antibody titer was measured by ELISA using (QGPGAP)_4_ peptide on day 33, and mice were challenged with infectious P. yoelii sporozoites intravenously on day 35. Cryopreserved P. yoelii sporozoites were purchased from Sanaria, Inc. (Rockville, MD), thawed immediately prior to challenge, and diluted according to instructions provided by Sanaria in order to deliver a challenge dose of 1,000 sporozoites/animal. Parasitemia was determined on day 7 and day 14 postchallenge by using a Giemsa-stained thin blood smear procedure. The investigators were blinded to allocation during the outcome assessment. Furthermore, 14 days after the challenge, blood was collected, and DNA was extracted by using a DNeasy blood and tissue kit (Qiagen, Hilden, Germany) and subjected to PCR analysis. Infection was determined by amplifying P. yoelii-specific 18S rRNA, and the mouse glyceraldehyde 3-phosphate dehydrogenase (GAPDH) gene was amplified as an internal control. The primers used were as follows: P. yoelii 18S rRNA, 5′-ACATGGCTATGACGGGTAACG and 5′-CCTTCCTTAGATGTGGTAGCTATTTCTC; mouse GAPDH, 5′-ACCACAGTCCATGCCATCAC and 5′-TCCACCACCCTGTTGCTGTA. PCR products were resolved on 2% agarose gels and visualized with SYBR Safe DNA gel stain (Thermo Fisher Scientific).

### Animal studies and statistical analysis.

No statistical methods were used to predetermine animal sample size. The samples sizes were chosen empirically to determine whether there were differences between samples. The experiments were not randomized. No animals were excluded from analysis. Statistical analyses were performed using Prism (GraphPad Software, La Jolla, CA). A Student *t* test was used for pairwise comparisons, and one-way analysis of variance (ANOVA) with Dunnett's multiple-comparison test was used for comparisons across multiple groups. For the quantitative analysis of VLP staining in mouse spleen, a Mann-Whitney U test was used. All statistical tests were two-tailed. *P* values of <0.05 were considered statistically significant.

## Supplementary Material

Supplemental material
