# Supplementary material for: Development of a Novel Virus-Like Particle Vaccine Platform That Mimics the Immature Form of Alphavirus
Source: Clin Vaccine Immunol. 2017 Jul 5;24(7):e00090-17. doi: 10.1128/CVI.00090-17 (PMC5498722; doi:10.1128/CVI.00090-17)
Supplement: Supplemental material [file supp_24_7_e00090-17__index.html]

Supplemental material 

# Development of a Novel Virus-Like Particle Vaccine Platform That Mimics the Immature Form of Alphavirus

## Supplemental material

- Supplemental file 1 -

  Fig. S1. Construction of αVLP expression vectors, and schematic representation of the VLPs constructed. Fig. S2. Validation of antibodies. Fig. S3. NANP repeat number optimization. Fig. S4. VLPM01-immunized monkey sera reduced liver parasite burden in mice.

  PDF, 428K
