# Supplementary material for: Development of a Novel Virus-Like Particle Vaccine Platform That Mimics the Immature Form of Alphavirus
Source: Clin Vaccine Immunol. 2017 Jul 5;24(7):e00090-17. doi: 10.1128/CVI.00090-17 (PMC5498722; doi:10.1128/CVI.00090-17)
Supplement: Supplemental material [file CVI.00090-17_zcd999095492s1.pdf]

FIG. S1

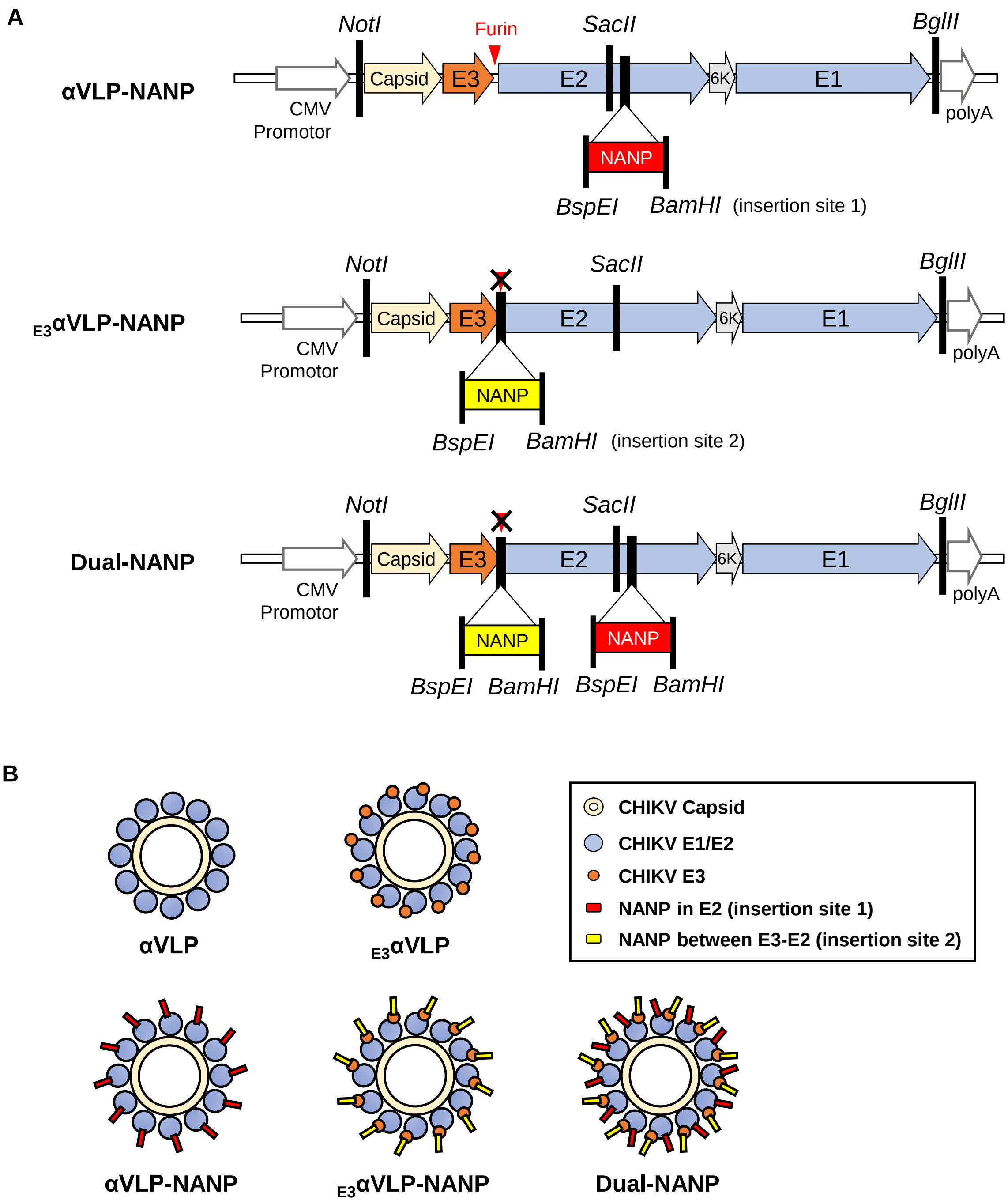

FIG. S1. (A) Construction of αVLP-expression vectors. A gene coding for CHIKV strain 37997 structural proteins (Capsid-E3-E2-6K-E1) was cloned under the control of human CMV early immediate promoter, and the epitope insertions sites were created by inserting *BspEI* and *BamHI* restriction sites as linkers. Furin recognition site in $_{E3}$αVLP is mutated, therefore $_{E3}$αVLP retains E3 on the VLP particle. *SacII* restriction site was introduced to create Dual-NANP. Dual-NANP was prepared by replacing *NotI/SacII* region of αVLP-NANP with *NotI/SacII* fragment of $_{E3}$αVLP-NANP. (B) Schematic representation of the VLPs constructed. αVLP-NANP and $_{E3}$αVLP-NANP display 240 copies of the inserted epitope. Dual-NANP displays 480 copies of the inserted epitope. VLPM01 and Daul-PyCSP use Dual construct and display 480 copies of the inserted epitopes.

FIG. S2

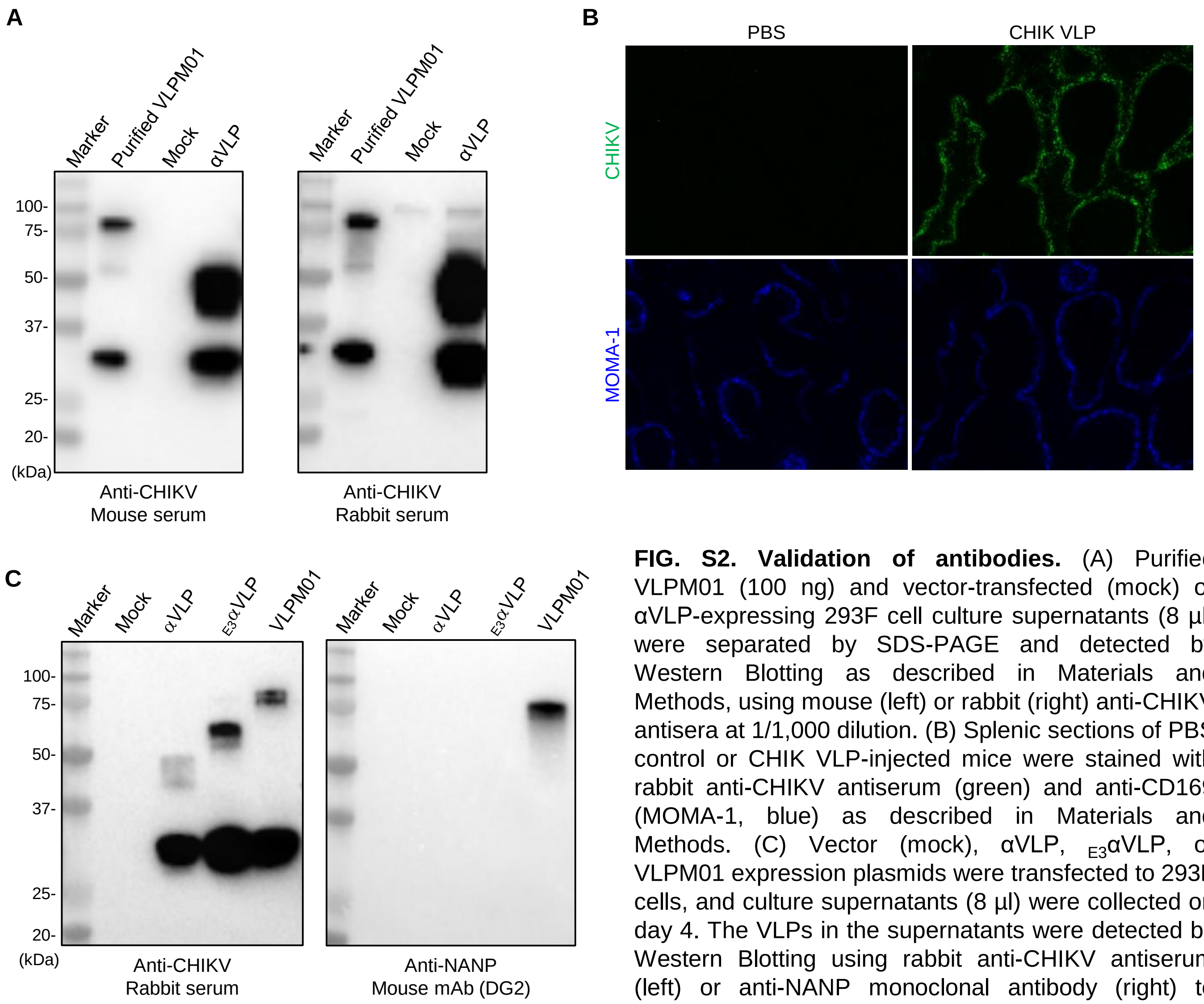

FIG. S2. Validation of antibodies. (A) Purified VLPM01 (100 ng) and vector-transfected (mock) or αVLP-expressing 293F cell culture supernatants (8 µl) were separated by SDS-PAGE and detected by Western Blotting as described in Materials and Methods, using mouse (left) or rabbit (right) anti-CHIKV antisera at 1/1,000 dilution. (B) Splenic sections of PBS control or CHIK VLP-injected mice were stained with rabbit anti-CHIKV antiserum (green) and anti-CD169 (MOMA-1, blue) as described in Materials and Methods. (C) Vector (mock), αVLP, $_{E3}$αVLP, or VLPM01 expression plasmids were transfected to 293F cells, and culture supernatants (8 µl) were collected on day 4. The VLPs in the supernatants were detected by Western Blotting using rabbit anti-CHIKV antiserum (left) or anti-NANP monoclonal antibody (right) to confirm the antibody specificity.

FIG. S3

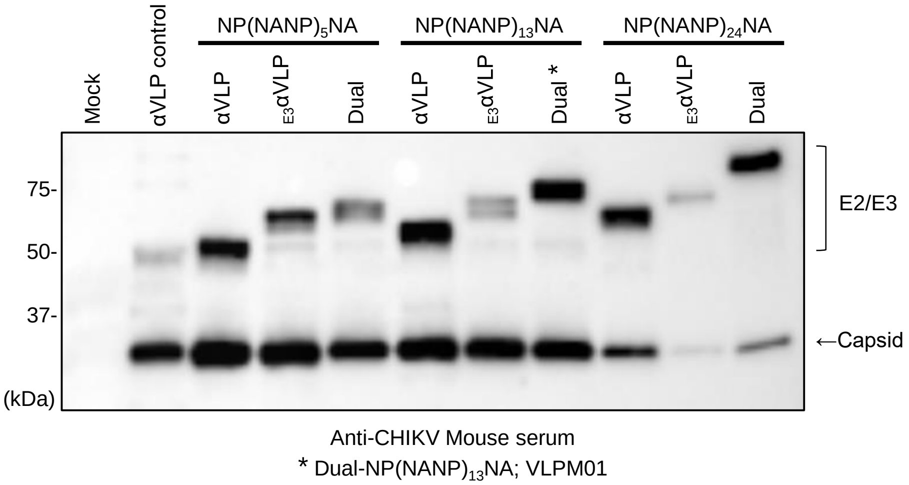

FIG. S3. NANP repeat number optimization. NANP repeat antigens were inserted into the VLP's insertion site 1 (αVLP), insertion site 2 ($_{E3}$αVLP), or both (Dual) as indicated in Fig. 3A. The VLPs were expressed in 293F cells, and the culture supernatants (8 µl) were collected on day 4. The VLPs in the supernatants were detected by Western Blotting using mouse anti-CHIKV antiserum. Mock; vector-transfected, αVLP control; αVLP without epitope.

FIG. S4

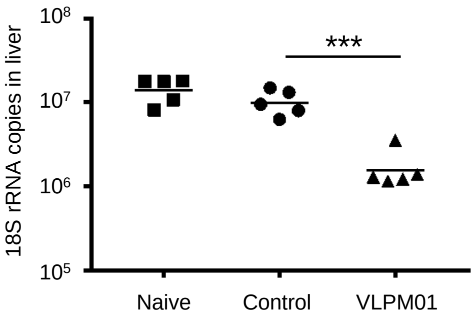

FIG. S4. VLPM01-immunized monkey sera reduced liver parasite burden in mice. B6 mice were injected intravenously with 450 µl of control or VLPM01-immunized monkey sera, then challenged intravenously with *Pb/Pf* full CSP sporozoites as described in Fig. 5C. Liver parasite burdens were assessed 40 hr post-challenge by qPCR for *P. berghei*-specific 18S rRNA. Plotted are values for individual mice (N=5) and the geometric mean of each group. Naïve mice did not receive monkey sera and were challenged with sporozoites. Student's t-test, where ***P < 0.001.
